# Evaluation of Five Non-Culture-Based Methods for the Diagnosis of Meningeal Sporotrichosis

**DOI:** 10.3390/jof9050535

**Published:** 2023-04-30

**Authors:** Fernando Almeida-Silva, Marcos de Abreu Almeida, Vanessa Brito de Souza Rabello, Rosely Maria Zancopé-Oliveira, Lilian Cristiane Baeza, Cristiane da Cruz Lamas, Marco Antonio Lima, Priscila Marques de Macedo, Maria Clara Gutierrez-Galhardo, Rodrigo Almeida-Paes, Dayvison Francis Saraiva Freitas

**Affiliations:** 1 Laboratório de Micologia, Instituto Nacional de Infectologia Evandro Chagas, Fiocruz, Rio de Janeiro 21040-360, RJ, Brazil; 2 Centro de Ciências Médicas e Farmacêuticas, Universidade Estadual do Oeste do Paraná, Cascavel 85819-110, PR, Brazil; 3 Serviço Médico, Instituto Nacional de Infectologia Evandro Chagas, Fiocruz, Rio de Janeiro 21040-360, RJ, Brazil; 4 Laboratório de Pesquisa Clínica em Neuroinfecções, Instituto Nacional de Infectologia Evandro Chagas, Fiocruz, Rio de Janeiro 21040-360, RJ, Brazil; 5 Laboratório de Pesquisa Clínica em Dermatologia Infecciosa, Instituto Nacional de Infectologia Evandro Chagas, Fiocruz, Rio de Janeiro 21040-360, RJ, Brazil

**Keywords:** *Sporothrix*, cerebrospinal fluid, molecular diagnosis, immunological diagnosis, qPCR, ELISA

## Abstract

Sporotrichosis is the main subcutaneous mycosis worldwide. Several complications, including meningeal forms, can be observed in immunocompromised individuals. The sporotrichosis diagnosis is time-consuming due to the culture’s limitations. The low fungal burden in cerebrospinal fluid (CSF) samples is another important drawback in the diagnosis of meningeal sporotrichosis. Molecular and immunological tests can improve the detection of *Sporothrix* spp. in clinical specimens. Therefore, the following five non-culture-based methods were evaluated for the detection of *Sporothrix* spp. in 30 CSF samples: (i) species-specific polymerase chain reaction (PCR); (ii) nested PCR; (iii) quantitative PCR; (iv) enzyme-linked immunosorbent assay (ELISA) for IgG detection; and (v) ELISA for IgM detection. The species-specific PCR was unsuccessful in the diagnosis of the meningeal sporotrichosis. The other four methods presented substantial levels of sensitivity (78.6% to 92.9%) and specificity (75% to 100%) for the indirect detection of *Sporothrix* spp. Both DNA-based methods presented similar accuracy (84.6%). Both ELISA methods were concomitantly positive only for patients with sporotrichosis and clinical signs of meningitis. We suggest that these methods should be implemented in clinical practice to detect *Sporothrix* spp. in CSF early, which may optimize treatment, augment the chances of a cure, and improve the prognosis of affected individuals.

## 1. Introduction

The central nervous system (CNS) involvement by fungal species is one of the most serious complications of the systemic mycoses. The *Cryptococcus neoformans*/*gattii* complex species are the main agents of fungal meningitis, occurring in both immunocompetent and immunocompromised individuals [1]. Histoplasmosis, caused by the globally distributed dimorphic fungus, *Histoplasma capsulatum*, may lead to CNS involvement in cases of immunosuppression [2]. The CNS involvement in coccidioidomycosis occurs with coccidioidal spherules or endospores migrating to the meninges or into the brain tissue [3]. The pathogenic dimorphic fungi belonging to the genus *Paracoccidioides* can cause neuroparacoccidioidomycosis, an uncommon but severe presentation of paracoccidioidomycosis [4]. Several other fungal species can cause CNS infection such as *Candida* spp., *Trichosporon* spp., *Aspergillus* spp., *Fusarium* spp., *Mucor* spp., *Rhizopus* spp., *Blastomyces dermatitidis*, *Cladophialophora bantiana*, and *Exophiala* dermatitidis, which may present fatal consequences [1].

Sporotrichosis is the most common subcutaneous mycosis, representing a global distribution. It is highly predominant in regions with tropical/subtropical climate and reached hyperendemic levels in several parts of Brazil, especially in Rio de Janeiro state. In this area, few cases are associated with trauma involving organic matter, whereas the zoonotic transmission prevails, notably due to the species *Sporothrix brasiliensis*, driving the increasing number of sporotrichosis cases in humans and cats [5].

Complications in sporotrichosis have been mostly seen and described in people living with HIV/AIDS (PLHIV), in whom the disease presents a poor prognosis. In addition, they were also described in other immunosuppressive conditions, such as diabetes and alcohol abuse [6]. Meningeal sporotrichosis is one of the most important complications in PLHIV and cases have been observed during the increase in sporotrichosis cases in Rio de Janeiro state since 1998 [7]. The virulence of *S. brasiliensis* is usually high, and it is suggested that this species may present neurotropism in immunosuppressive settings, which occurs in PLHIV, due to failure in antiretroviral therapy, low adherence to treatment, or the unawareness of their HIV status [7,8,9].

Culture isolation of *Sporothrix* spp. is the gold standard for the sporotrichosis diagnosis, regardless of its clinical manifestations. However, this method is time-consuming and lacks sensitivity in clinical materials with an associated microbiota or in cases of low fungal burden [10], which frequently occurs in the cerebrospinal fluid (CSF), making culture very limiting for the diagnosis of meningeal sporotrichosis. Currently, several other methods have been developed to improve diagnosis. DNA-based methods in the clinical practice are used for several purposes, especially to identify etiologic agents. Molecular methods improve both identification and diagnosis of fungal infections due to high sensitivity and specificity in several clinical specimens in which gold standard tests present intrinsic limitations. Nevertheless, molecular methods only offer a presumptive diagnosis due to, for instance, the inability to differentiate between colonization and infection or between live and dead fungal cells [11]. Immunoassays are also important alternative tools in the diagnosis of sporotrichosis, especially in extracutaneous manifestations, which often require invasive procedures to obtain biological samples for culture [12]. In addition to the fast turnaround time, these tests have excellent levels of sensitivity and, in some cases, can be even used for a therapeutic follow-up [13].

In fact, serological and molecular methods have presented good performances for the sporotrichosis diagnosis in some clinical samples, but few studies have been applied to CSF samples up to the present time [14]. Methods that detect the fungus or its biomarkers in the CSF would greatly improve the diagnosis of meningeal sporotrichosis. The aim of this study was to evaluate the performance of five non-culture-based methods available in the literature with potential use in CSF samples to provide reliable and fast results for the diagnosis of meningeal sporotrichosis.

## 2. Materials and Methods

### 2.1. Study Location, Design, and Samples

Two cross-sectional studies, approved by the Institutional Review Board (#88551018.9.0000.5262 and #54249721.9.0000.5262), were carried out at the Instituto Nacional de Infectologia Evandro Chagas, Fundação Oswaldo Cruz (INI/FIOCRUZ), a reference center for infectious diseases, including sporotrichosis in the hyperendemic area of Rio de Janeiro, Brazil.

We included 14 CSF samples from six patients with disseminated sporotrichosis and meningitis, hereinafter referred to as the case group. One patient of this group had one sample, two patients had two different samples and three had three different samples, collected at distinct times. In addition, 12 CSF samples from 12 patients with other diseases, infectious or not, were used as controls (hereinafter referred to as the control group). Finally, four CSF samples from four patients with disseminated sporotrichosis, but without diagnostic criteria for meningitis, were additionally investigated to check whether *Sporothrix* spp. would be involved in asymptomatic meningitis (hereinafter referred to as the test group). All patients underwent lumbar puncture for a routine CNS infection investigation in the study place, using good medical practices.

### 2.2. Definitions

Sporotrichosis was defined as the development of clinical signs and/or symptoms in a patient with the isolation of *Sporothrix* sp. In culture from any clinical specimen.

Meningeal sporotrichosis was defined as the *Sporothrix* sp. Isolation in culture from the CSF or as the detection of biochemical or cytological alterations in the CSF in patients with culture-proven sporotrichosis from any other site and with no other confirmed neurological cause.

### 2.3. Routine CSF Analysis

The institutional routine for CSF analyses included biochemistry, cytology, nontreponemal syphilis evaluation (VDRL), lateral flow assay for cryptococcal antigen, GeneXpert for *Mycobacterium tuberculosis*/resistance to rifampin (MTB/RIF), acid-fast bacilli detection, specific cultures for mycobacteria, regular bacteria, and fungi, multiplex polymerase chain reaction (PCR) for human herpesviruses (herpes simplex viruses 1 and 2, varicella-zoster virus, Epstein-Barr virus, cytomegalovirus, and human herpesviruses 6 and 8), and PCR for the John Cunningham (JC) virus [7].

### 2.4. DNA Extraction and Quality Controls

The DNA extraction was performed with the QIAamp DNA mini kit (QIAGEN, Hilden, Germany) using 200 μL of the CSF samples previously centrifuged at 13,000× *g*, according to the manufacturer’s instructions. Extracted DNA was quantified using the Biophotometer Plus (Eppendorf, Hamburg, Germany) equipment. DNA samples were maintained at −20 °C until use. The 79 base pairs (bp) fragment of human β-globin gene was amplified, using 50 p-moles of primers, 5′-GCAAGAAAGTGCTCGGTGC-3′ (Forward) and 5′-TCACTCAGTGTGGCAAAGGTG-3′ (Reverse), and 2.5 p-moles of probe (5′-FAM-TAGTGATGGCCTGGCTCACCTGGAC-3′-TAMRA) aiming to evaluate potential PCR inhibitors, using the Agilent AriaMX qPCR thermal cycler (Agilent Technologies, Santa Clara, CA, USA)**,** as previously described [15]. Samples without DNA amplification were excluded for the subsequent analyses. Another control test involved genomic DNA extracted from fungal species related to CNS infections, such as *Histoplasma capsulatum* (ATCC 26032), *Trichosporon asahii* (isolate 53897), *Candida albicans* (ATCC 18804), *Cryptococcus neoformans* (ATCC 208821) and *Cryptococcus gattii* (ATCC 56990), as previously described [16].

### 2.5. Diagnostic Methods

#### 2.5.1. DNA-Based Methods

Three molecular methods were performed for the *Sporothrix* detection in extracted DNA from CSF using the QIAamp DNA mini kit (QIAGEN, Hilden, Germany).

##### Nested PCR

Firstly, a nested PCR targeting the 18S rRNA gene [17] was performed with the primers SS1 (5′-CTCGTTCGGCACCTTACACG-3′) and SS2 (5′-CGCTGCCAAAGCAACGCGGG-3′) and in the second reaction, the primers SS3 (5′-ACTCACCAGGTCCAGACACGATG-3′) and SS4 (5′-CGCGGGCTATTTAGCAGGTTAAG-3′), were used to amplify a 152 bp fragment. The reaction mixture (25 µL) consisted in final concentrations of 10 mM Tris-HCl (pH 9.0), 50 mM KCl, 1.5 mM MgCl_2_, 0.4 µM of each primer, 1.5 U of *Taq* DNA polymerase (Invitrogen, Waltham, MA, USA), and 200 µM of each dNTP (Invitrogen). The template for the first reaction consisted of 4 µL of total DNA extracted from CSF and, for the second, 4 µL of the products amplified in the first reaction. Both cycles were performed in a T100 thermal cycler (Bio-Rad Laboratories Inc., Hercules, CA, USA), as follows: 95 °C for 5 min, 35 cycles at 95 °C for 30 s, 60 °C for 45 s, 72 °C for 30 s, and a final extension of 72 °C for 10 min. The DNA of the reference strain *S. brasiliensis* (CBS 120339) was included as positive control, and the negative control was performed substituting the DNA with the same volume of ultrapure water (Gibco, Walthan, MA, USA).

##### Species-Specific PCR

The species-specific PCR for *S. brasiliensis*, using the primers Sbra-F→(5′-CCCCCGTTTGACGCTTGG-3′) and Sbra-R (5′-CCCGGATAACCGTGTGTCATAAT-3′), was performed according to a previously described test [18], aiming to amplify a fragment of 469 bp. The species-specific PCR was performed in a T100 thermal cycler (Bio-Rad Laboratories Inc., Hercules, CA, USA), using 25 µL as final volume, and the amplified fragments were compared with the 1 kb plus DNA ladder (Invitrogen) in a 1% electrophoresis agarose gel stained with 0.5% ethidium bromide.

##### Multiplex qPCR

A quantitative PCR (qPCR) aimed at *S. brasiliensis* detection was performed as previously described [19], with minor modifications. It used 10 µm of each primer (Spo-F 5′-CATTGACTTCCCTGGTAYGTTTGAC-3′ and Spo-R 5′-CARGAACTCTGTGGAYGGTTAGC-3′). However, the probe previously named as Spo-MGB-SBP was modified to NED-ACACACGGTTATCC-MGB. The reactions, using 20 µL of final volume, were performed in the Agilent AriaMX qPCR thermal cycler (Agilent Technologies, Santa Clara, CA, USA), and consisted of a polymerase activation at 95 °C for 2 min and 45 cycles of annealing/extension at 60 °C for 50 s. The results were analyzed in the Aria-MX software (version 1.7.1).

#### 2.5.2. Limits of Detection (LOD)

To simulate the conditions of meningeal sporotrichosis, serial 10-fold dilutions of *S. brasiliensis* (CBS 120339) DNA were performed in artificial CSF (127 mM NaCl, 1.0 mM KCl, 1.2 mM KH_2_PO_4_, 26 mM NaHCO_3_, 10 mM D-glucose, 2.4 mM CaCl_2_, 1.3 mM MgCl_2_, pH 7.0). Dilutions ranging from 100 ng/µL to 1 fg/µL of DNA were used to test the sensitivity of each molecular method. The detection limit was defined as the lowest concentration of target DNA detected by each method.

#### 2.5.3. Immunoassays

Indirect enzyme-linked immunosorbent assay (ELISA), in two different formats, was performed, as described previously [20], with slight modifications to detect IgG and IgM class antibodies against *Sporothrix* spp. Optimization of our ELISA applied to serum samples was performed for the use of CSF as a biological sample. CSF samples were diluted from 1:2 to 1:32,000 and the best dilution to discriminate between positive and negative was 1:100. Following optimization, the ELISAs were used to evaluate the CSF samples. This experiment was performed in triplicate, and the ELISA cutoff point was calculated as the mean optical densities (ODs) plus three standard deviations of the controls. ODs above the cutoff were considered positive.

##### ELISA for IgG detection

For IgG detection, goat anti-human IgG peroxidase conjugate (Jackson Immunoresearch Laboratories, West Grove, PA, USA) was used as the secondary antibody diluted 1:32,000 in incubation buffer (10 mM PBS, 0.1% Tween 20, 5% nonfat skimmed milk powder [pH 7.2]). The enzymatic reaction for IgG detection was developed with the addition of 100 μL per well of 0.4 mg of o-phenylenediamine dihydrochloride (OPD)/mL and 0.04% hydrogen peroxide in 10 mM sodium citrate buffer (pH 5.5). The reaction was stopped by the addition of 50 μL of 3 M HCl per well. Absorbances were measured using the SpectraMax Plus spectrophotometer (Molecular Devices, San Jose, CA, USA) at 490 nm.

##### ELISA for IgM detection

The secondary antibody for IgM detection was goat anti-human IgM alkaline phosphatase conjugate (Southern Biotech, Birmingham, UK) diluted 1:2000 in 10 mM TBS, 0.1% Tween 20, and 5% nonfat skimmed milk powder (pH 7.2) at a final volume of 100 μL per well. The enzymatic reaction was developed with the addition of 100 μL per well of 1.0 mg of p-nitrophenyl phosphate (PNPP)/mL in 0.1 M glycine buffer containing 1 mM MgCl_2_ and 1 mM ZnCl_2_ (pH 10.4), and the reaction was stopped by the addition of 25 μL of 3 M NaOH per well. The same Spectra Max Plus spectrophotometer (Molecular Devices, San Jose, CA, USA), at 405 nm, was used to measure the absorbances in this reaction.

### 2.6. Data Analysis

Clinical performance of these methods in the population of patients with meningeal sporotrichosis and other CNS diseases was assessed using 2 × 2 tables to calculate sensitivity, specificity, accuracy, and positive and negative likelihood ratios using the MedCalc Software Ltd., Ostend, Belgium. Diagnostic test evaluation calculator, version 20.211, freely available at https://www.medcalc.org/calc/diagnostic_test.php (accessed on 08 March 2023).

## 3. Results

### 3.1. Patients

The six patients with meningeal sporotrichosis (case group) were males from the metropolitan region of Rio de Janeiro, with a median age of 40 (range: 25–57) years. Among them, four mentioned previous contact with a sick cat suspected of sporotrichosis, and one reported scratches from a cat with sporotrichosis. The other patient was a gardener who was in daily contact with soil. All patients were PLHIV, with a median CD4+ T lymphocyte count of 104.5 (range: 29–302) cells/μL at the first lumbar puncture. By the end of this analysis, four patients had died due to sporotrichosis or other AIDS-related conditions, and two patients were cured of sporotrichosis.

In the control group (patients without sporotrichosis), eleven were males and one was female, being nine PLHIV, with a median age of 41 (range: 30–66) years. Six patients had distinct CNS infections, as follows: meningeal tuberculosis, cryptococcal meningitis, neuroparacoccidioidomycosis, varicella-zoster virus, cytomegalovirus with herpes simplex 2 virus, and Epstein Barr virus, one patient each. Among the other six patients, four were PLHIV with neurological symptoms, one patient had a headache, and another presented a posterior uveitis, all with no specific neurological diagnosis.

In the test group (four patients with sporotrichosis without criteria for meningitis after the routine investigation), three were males and one was female. Their median age was 42 (range 35–66) years and they also lived in the metropolitan region of Rio de Janeiro. Two reported contact with a sick cat and one of them was scratched. The other two patients could not remember any risk activities for sporotrichosis. Three patients were PLHIV, with a median CD4+ T lymphocyte count of 88 (range: 77–197) cells/μL and two of them are currently cured of sporotrichosis, while the woman was lost to follow-up. The fourth patient had uncontrolled diabetes and died due to sporotrichosis complications.

### 3.2. DNA-Based Methods

Quality control tests revealed amplification of 79 bp fragments in all 30 CSF samples, using the qPCR (β-globin-FAM-TAMRA). Three other potential samples were excluded due to the absence of beta-globin amplification and were not submitted to the methods. The DNA samples extracted from fungal species related to CNS infections were negative in all three studied DNA-based methods. The LOD of the nested PCR, qPCR, and species-specific PCR methods were 10^−2^ pg/µL, 10^2^ pg/µL, and 10^5^ pg/µL, respectively.

**Figure 1:**
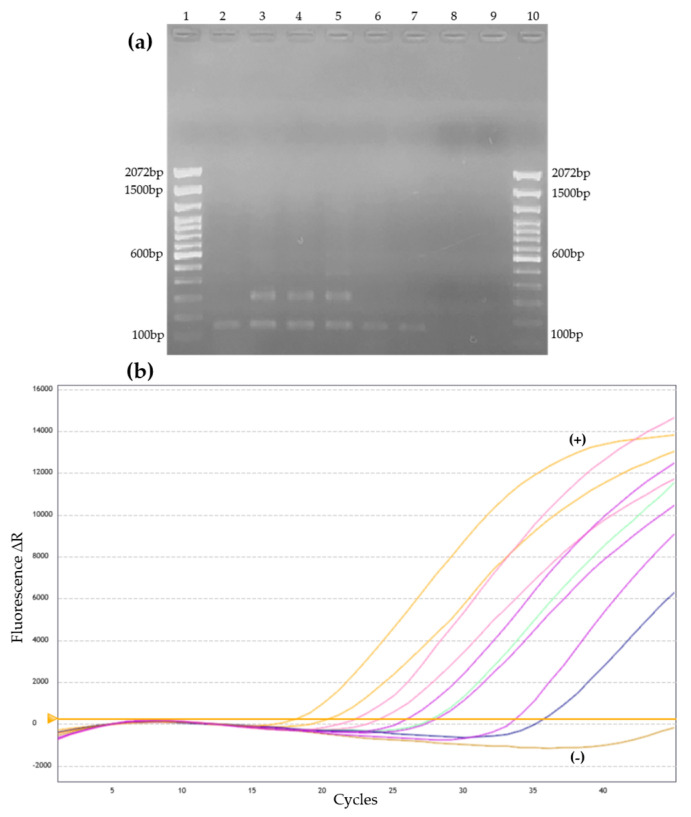
Representative amplification profile of CSF samples in two DNA-based methods for *Sporothrix* spp. detection. (**a**) Nested PCR-Agarose gel with positive CSF samples, demonstrating 152 bp fragments compatible with *Sporothrix* spp. Values 1 and 10 = Molecular weight (100 pb Plus–Invitrogen), 2 to 6 = positive CSF samples, 7 = positive control of DNA extracted from *Sporothrix brasiliensis* culture (CBS 120339), 8 = PCR mix negative control, and 9 = PCR mix negative control with water addition. (**b**) Amplification curves of the qPCR observed in positive CSF samples using the CY3 fluorescence channel. Each colored curve represents a different sample. The yellow horizontal line represents the threshold of the reaction. +: positive control. −: negative control.

Among the DNA-based methods herein studied, the nested PCR was the method with the highest number of positive samples. This method detected 19 positive samples, 13 in the case group, and three positive samples in each one of the other two groups. The qPCR detected 13 positive samples, 11 in the case group, and one in each of the other two groups. All samples were negative in the *Sporothrix* spp. species-specific PCR. Figure 1 demonstrates a representative profile of amplification in the nested PCR and qPCR.

### 3.3. Immunoassays

**Figure 2:**
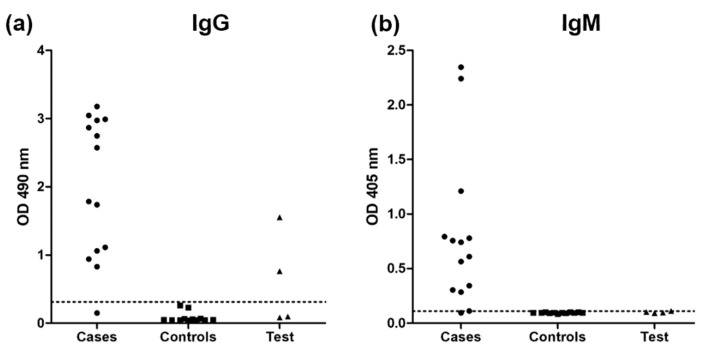
Detection by enzyme-linked immunosorbent assay (ELISA) of (**a**) IgG and (**b**) IgM responses against the mycelial phase *S. brasiliensis* exoantigens in cerebrospinal fluid samples from patients with disseminated sporotrichosis and meningitis (cases), patients without sporotrichosis (controls), and patients with sporotrichosis but without criteria for meningitis (tests). The doted horizontal lines indicate the cutoff values for each single ELISA.

The ELISA for the detection of IgG antibodies was reactive in 13/14 CSF samples from the case group and in 3/4 samples from patients in the test group, while 12/12 samples from the control group were negative. When the ELISA was used for IgM detection, similar results were found, with 13 reactive samples from the case group, one reactive sample from a patient from the test group, and no reactivity in control samples. Figure 2 presents the ODs of each sample evaluated within the three groups.

### 3.4. Diagnostic Performance of the Methods

Table 1 summarizes the results obtained for each CSF sample using the four different non-culture-based methods evaluated that presented positive results.

Table 2 presents sensitivity, specificity, accuracy, and likelihood ratios of the four methods which presented positive results herein studied. Among DNA-based methods, the nested PCR presented higher sensitivity (92.9%), but lower specificity (75%). The serological parameters observed for both IgG and IgM detection showed similar results; both showing sensitivity equal to the nested PCR and specificity higher than any DNA-based test.

## 4. Discussion

Sporotrichosis is an infection of a benign course in the majority of the cases, restricted to the skin [9]. In immunocompromised patients, sporotrichosis can disseminate to several organs and one of the most important complications is the dissemination to the CNS, with unfavorable prognosis and high mortality [21]. The sporotrichosis chronic meningitis is indistinguishable from other etiologies. Moreover, it was recently demonstrated that approximately 40% of patients with meningeal sporotrichosis due to *S. brasiliensis*, from a cohort of 17 cases, did not present neurological symptoms at the first lumbar puncture, which were seen later. When we compared these patients with meningeal sporotrichosis (n = 17) vs. cutaneous-disseminated/disseminated sporotrichosis without meningitis (n = 36), lethality among patients with meningeal sporotrichosis was higher (64.7%), with a higher chance of death (HR = 3.87) [7]. Early asymptomatic meningeal inflammation associated with low fungal burden in CSF may delay its diagnosis and compromise the prognosis of patients [7,22]. Some methods presented here, both DNA-based and immunoassays, have potential to detect presumptively *Sporothrix* spp., possibly making the prognosis of these patients more favorable.

Several diagnostic methods can overcome the limitations of gold-standard diagnosis. The increase in sensitivity and specificity values are the main important advantages of the DNA-based methods and immunoassays. Other important advantages of these assays are the reduced turnaround time, which is associated with an improvement in the quality of life of affected individuals [23]. The combination of DNA-based methods and immunoassays is frequently used to diagnose fungal infections, such as histoplasmosis [24], aspergillosis [25], and fungal coinfections in immunocompromised patients [26]. This combination of methods, as the results herein suggest, is a feasible and important tool for the diagnosis of meningeal sporotrichosis, which had not yet been demonstrated for the CSF.

In sporotrichosis, different diagnostic DNA-based methods are reported. The species-specific PCR was developed for species identification [18], but can be used for *Sporothrix* DNA detection in clinical samples, such as biopsy [19], and in soil samples [27]. However, this technique did not demonstrate positive results for the CSF samples herein analyzed, even those with *S. brasiliensis* isolation in culture, which can be attributed to the lower limit of detection of this method, associated to the low fungal burden expected to be found in CSF samples from patients with meningeal sporotrichosis [22].

Nested PCR and qPCR are reported as methods with higher sensitivity and specificity. For several fungal infections, these methods are used as diagnostic tests in the laboratorial routine [28,29], but few are applied for sporotrichosis [30]. Although qPCR presented lower sensitivity than nested PCR in our study, it proved to be a highly specific technique, which provides evidence for its usefulness for the diagnosis of meningeal sporotrichosis due to *S. brasiliensis*. The false negative result for the nested PCR of sample 2a, from which there was the isolation of *S. brasiliensis* in culture, is something to be answered. Perhaps the analysis of more samples will help the understanding of what we currently believe to be a limitation of the method. The negative results for the qPCR of samples 3a and 3b may also be a limitation of the method, which, in this case, can be partially explained by the lower LOD when compared to the nested PCR. Currently, *S. brasiliensis* is the main agent of meningeal sporotrichosis in Brazil [7], with a potential to spread to other countries [31]. Furthermore, the diagnosis of meningeal sporotrichosis due to other *Sporothrix* species could benefit from the same technique, following the necessary modifications.

In a single-center study, a nested PCR for fungi was successfully used as a diagnostic test in 50 clinical samples, with a better performance than culture (21/50 vs. 0/50) [29]. The nested PCR presented a higher number of positive samples. Recently, this method was applied in environmental samples, aiming to detect *Sporothrix* spp., but demonstrated unspecific amplification, detecting several fungi of the Ophiostomataceae family after sequencing [27]. Most of these fungi are not pathogenic for mammals, only saprobic from organic matter. Based on the current knowledge, there are no other members of this fungal family, besides the pathogenic *Sporothrix* spp., that can infect the CNS, which supports that, in the CSF, this nested PCR detects pathogenic species of the genus *Sporothrix*. Thus, the nested PCR can be used in the diagnosis of sporotrichosis, but carefully. As this test presented the lower specificity among the methodologies used, its use without other tests could lead to a misdiagnosis of meningeal sporotrichosis in patients without this condition, leading to erroneous medication administration and possible unfavorable outcomes. Moreover, an important universal precaution to be taken when performing nested PCR is to avoid cross-contamination, which has high odds of occurring between the first and second reactions [32].

The ELISA test and the antigen used in this study were previously analyzed in serum samples for IgG detection, with substantial sensitivity and specificity values (97% and 89%, respectively) [20]. The same test was further optimized for the detection of IgM and IgA in serum from patients with sporotrichosis, suggesting that the detection of combined IgA, IgG, and IgM antibodies is a highly sensitive and specific diagnostic assay for sporotrichosis [33]. Although no antibody detection test has been validated in the CSF of patients with meningitis caused by *Sporothrix* spp., some studies demonstrate the usefulness of these tools in the diagnosis of sporotrichosis affecting the CNS [14,34]. Using an ELISA test, Scott and colleagues (1987) [14] described for the first time the feasibility of antibody detection in the CSF of patients with sporotrichosis. The aforementioned work also reports the use of the latex agglutination test as another technique capable of detecting anti-*Sporothrix* antibodies in a CSF sample. Additionally, the literature also reports the possibility of using the detection of anti-*Sporothrix* antibodies in CSF to monitor patients with meningeal sporotrichosis. Some studies describe an increase in the level of antibodies in the CSF in episodes of recurrence and a decrease with clinical improvement [14,34].

Our results also showed that it is important to evaluate both IgM and IgG responses, because patients with sporotrichosis, but without meningeal sporotrichosis, may present IgG antibodies in the CSF, possibly due to the antibody crossing the blood-brain barrier.

Some samples had different results from the expected. In sample 5, the negativity in both ELISAs can be explained by a lack of immune response in a PLHIV with comorbidities and abandonment of treatment. Among the controls, sample 9 was from an undiagnosed patient in our institute. The most probable is that a cross-reactivity, which occurs in very sensitive techniques, may have happened, because the clinical picture was not compatible with sporotrichosis. Among the samples from the four patients with sporotrichosis but without clinical criteria for meningitis, none had concomitant positive IgG and IgM results in the CSF sample. Sample 19 had isolated positive IgG in the CSF. IgG may have crossed the blood-brain barrier. Samples 20 and 22 came from patients who did not have criteria for CNS involvement and were discharged from follow-up after sporotrichosis cure. Therefore, the isolated positive nested PCR for both seems nonspecific and, more likely, a false positive result. While in sample 21, the combination of positive nested PCR, qPCR, and IgG antibody in the CSF highlights the probability of the presence of the fungus in the CNS with no meningitis signs, in both clinical and laboratory settings. A hypothesis is that the fungus entered the CNS but did not manage to produce meningitis. It is interesting to note that this patient received treatment for disseminated sporotrichosis, with amphotericin B, until clinical improvement, followed by oral itraconazole [6]. By the last medical appointment, she was well, but lost follow-up. It is also important to note that all samples with confirmed other fungal, viral, or bacterial infections were negative for the five methods analyzed.

Some limitations of this study include: (i) small number of CSF samples, due to the fact that meningeal sporotrichosis is a rare clinical manifestation, (ii) evaluation of meningeal sporotrichosis caused only by *S. brasiliensis*, two cases with culture confirmation and four presumed through the qPCR result, making it difficult to know whether meningitis caused by other *Sporothrix* species could be diagnosed with the same efficiency, and (iii) the real prevalence of meningeal sporotrichosis is unknown, which hinders the calculation of predictive values.

## 5. Conclusions

Meningeal sporotrichosis is associated with poor prognosis, and better diagnosis strategies are needed to improve the approach of these patients. The combination of molecular and immunological methods can improve the diagnosis of meningeal sporotrichosis, overcoming the limitations of conventional diagnostic methods. According to our results, the species-specific PCR is not useful for this purpose. Immunoassays, together with qPCR, should be used as the first diagnostic tool due to the high chance of detecting a positive sample. Finally, nested PCR can be used, considering the necessary precautions during the performance of this technique, to further screen negative samples in the previous techniques or in places that cannot afford qPCR.

## Figures and Tables

**Table 1:** Summary of clinical aspects of the patients and results obtained with four different non-culture-based methods for the diagnosis of sporotrichosis using cerebrospinal fluid samples.

Patient Sample (CSF)		Diagnosis and Comorbidity/Immunosuppression	Routine Findings	Nested PCR	qPCR	ELISA	
						IgG	IgM
**^a^ Case group**	1a	Disseminated sporotrichosis/HIV/AIDS/Past of seizures (childhood)	Pleocytosis, low glucose and high protein	+	+	+	+
	1b		Pleocytosis and low glucose	+	+	+	+
	1c		Normal	+	+	+	+
	2a	Disseminated sporotrichosis/HIV/AIDS/Depression	Pleocytosis, low glucose, high protein and *Sporothrix* sp. in culture	−	+	+	+
	2b		Pleocytosis, low glucose, high protein and *Sporothrix* sp. in culture	+	+	+	+
	3a	Disseminated sporotrichosis/HIV/AIDS/Previous pulmonary TB	Pleocytosis, low glucose and high protein	+	−	+	+
	3b		Pleocytosis and high protein	+	−	+	+
	3c		Pleocytosis, low glucose and high protein	+	+	+	+
	4a	Disseminated sporotrichosis/HIV/AIDS/Previous syphilis	Pleocytosis, low glucose and high protein	+	+	+	+
	4b		Pleocytosis, low glucose and high protein	+	+	+	+
	4c		Pleocytosis, low glucose and high protein	+	−	+	+
	5	Disseminated sporotrichosis/HIV/AIDS/Disseminated TB/Alcohol abuse	*Sporothrix* sp. in culture	+	+	−	−
	6a	Disseminated sporotrichosis/HIV/AIDS/Asthma	Pleocytosis and high protein	+	+	+	+
	6b		High protein	+	+	+	+
**Control group**	7	Previous disseminated sporotrichosis/Meningeal TB/HIV/AIDS	High protein/ *Mycobacterium tuberculosis*	−	−	−	−
	8	Spinal syndrome/HIV/AIDS/Pulmonary TB/Syphilis/Disseminated histoplasmosis	Pleocytosis, EBV	−	−	−	−
	9	Posterior uveitis, optic disc edema	Normal	+	+	−	−
	10	Investigation of headaches	Normal	−	−	−	−
	11	Paracoccidioidomycosis/HIV/AIDS	Normal	−	−	−	−
	12	Disseminated histoplasmosis/Facial herpes zoster/HIV/AIDS	High protein/VZV	−	−	−	−
	13	Pansinusitis/HIV/AIDS	Normal	−	−	−	−
	14	PML/Disseminated TB/HIV/AIDS	HSV-2, CMV	−	−	−	−
	15	Neuroparacoccidioidomycosis	Normal	−	−	−	−
	16	COVID-19 and *Pneumocystis jirovecii* pneumonia/HIV/AIDS	Normal	+	−	−	−
	17	PML/HIV/AIDS	Normal	+	−	−	−
	18	Cryptococcal meningitis/Disseminated histoplasmosis/HIV/AIDS	Cryptococcal antigen positive	−	−	−	−
**^a^ Test group**	19	Disseminated sporotrichosis/DM	Normal	−	−	+	−
	20	Disseminated sporotrichosis/HIV/AIDS	Normal	+	−	−	−
	21	Disseminated sporotrichosis/Latent syphilis/Pulmonary TB/HIV/AIDS	Normal	+	+	+	−
	22	Disseminated sporotrichosis/Facial palsy and neuromotor impairment since birthday/HIV/AIDS	High protein	+	−	−	+

CSF: cerebrospinal fluid. HIV/AIDS: Infection by the human immunodeficiency virus with or without acquired immunodeficiency syndrome. COVID-19: Coronavirus disease 2019. PCR: polymerase chain reaction. qPCR: quantitative PCR. ELISA: enzyme-linked immunosorbent assay. NA: not available. DM: Diabetes mellitus. PML: Progressive multifocal leukoencephalopathy. HSV: Herpes simplex virus. CMV: cytomegalovirus. VZV: varicella-zoster virus. TB: tuberculosis. EBV: Epstein-Barr virus. +: positive or reactive results. −: negative or non-reactive results. ^a^—Detailed characteristics of the six patients from the case group were previously published by Lima et al. [7], as patients 1–2 and 13–16; patients from samples 20–22 of the test group were part of the Group 2 of the article by Lima et al. [7].

**Table 2:** Performance of four non-culture-based methods when testing cerebrospinal fluid samples from patients with meningeal sporotrichosis and with other suspected central nervous system diseases.

Parameter	Nested PCR	qPCR	IgG ELISA	IgM ELISA
Sensitivity	92.9% (66.1–99.8)	78.6% (49.2–95.3)	92.9% (66.1–99.8)	92.9% (66.1–99.8)
Specificity	75.0% (42.8–94.5)	91.7% (61.5–99.8)	100% (73.5–100)	100% (73.5–100)
Accuracy	84.6% (65.1–95.6)	84.6% (65.1–95.6)	96.2% (80.4–99.9)	96.2% (80.4–99.9)
Positive likelihood ratio	3.71 (1.38–10.00)	9.43 (1.42–62.81)	∞	∞
Negative likelihood ratio	0.10 (0.01–0.65)	0.23 (0.08–0.65)	0.07 (0.01–0.47)	0.07 (0.01–0.47)

PCR: polymerase chain reaction. qPCR: quantitative PCR. ELISA: enzyme-linked immunosorbent assay. The 95% confidence interval is presented within the parenthesis. ∞: infinity.

## Data Availability

All relevant data are presented in the article.
